# Early Responses to Intravitreal Ranibizumab in Typical Neovascular Age-Related Macular Degeneration and Polypoidal Choroidal Vasculopathy

**DOI:** 10.1155/2011/742020

**Published:** 2011-06-12

**Authors:** Wataru Matsumiya, Shigeru Honda, Hiroaki Bessho, Sentaro Kusuhara, Yasutomo Tsukahara, Akira Negi

**Affiliations:** Department of Surgery, Division of Ophthalmology, Kobe University Graduate School of Medicine, Kobe 650-0017, Japan

## Abstract

*Purpose*. To evaluate the early response to intravitreal ranibizumab (IVR) in two different phenotypes of age-related macular degenerations (AMD): typical neovascular AMD (tAMD) and polypoidal choroidal vasculopathy (PCV). *Methods*. Sixty eyes from 60 patients (tAMD 28, PCV 32 eyes) were recruited. Three consecutive IVR treatments (0.5 mg) were performed every month. Change in the best-corrected visual acuity (BCVA) and central retinal thickness (CRT) was then compared between the tAMD and PCV groups. *Results*. The mean BCVA logMAR was significantly improved at month 1 and month 3 after the initial IVR in the tAMD group, but there was no change in the PCV group. Both phenotypes showed significant improvements in the CRT during the 3 months after the initial IVR. There were no significant differences in the improvements of the CRT in the tAMD versus the PCV group. In the stepwise analysis, a worse pretreatment BCVA and tAMD lesions were significantly beneficial for a greater improvement of BCVA at 3 months after the initial IVR. *Conclusions*. The phenotype of tAMD showed a significantly better early response to IVR than PCV in terms of BCVA improvement.

## 1. Introduction

The intravitreal injection of ranibizumab (IVR) is currently the treatment of choice for subfoveal choroidal neovascularization (CNV) due to age-related macular degeneration (AMD), a leading cause of central visual loss in the elderly in industrialized countries [1, 2]. Several studies from Western countries have reported a significant improvement in vision at 3 months with monthly IVRs [3, 4]. However, the efficacy of IVR has not been investigated well for exudative AMD in the Japanese population. A recent report described a good response to intravitreous bevacizumab in Japanese AMD patients with classic CNV lesions, but there was limited efficacy in those with occult CNV lesion [5]. We hypothesized that those results might be attributed to the proportion of AMD subtypes in the Japanese population, which includes polypoidal choroidal vasculopathy (PCV) as the major phenotype of exudative AMD [6], and the effects of antivascular endothelial growth factor (VEGF) therapy for PCV may differ from those for typical neovascular AMD (tAMD). Recent publications have reported that the effects of anti-VEGF therapy were limited in PCV [7–9]. However, to our knowledge, no comparative studies have been published on the effectiveness of IVR associated with the different phenotypes of AMD.

In this study, we first performed a comparative assessment to determine whether the early responses to IVR were different between tAMD and PCV. In addition, a multiple regression analysis was performed to determine if the AMD phenotype (tAMD or PCV) may influence the visual outcomes of IVR independently, with several pretreatment factors which might affect the outcomes of the IVR.

## 2. Subjects and Methods

All cases in this study were Japanese individuals recruited from the Department of Ophthalmology at the Kobe University Hospital in Japan. Written informed consent was obtained from all subjects.

Sixty eyes from 60 patients (tAMD 28, PCV 32 eyes) were recruited. All patients received detailed ophthalmic examinations, including best-corrected visual acuity (BCVA) measurements, slit lamp biomicroscopy of their fundi, color fundus photography, optical coherence tomography, fluorescein angiography (FA), indocyanine green angiography (ICG), and optical coherence tomography (OCT). A detailed questionnaire on the patient's basic characteristics including age, body weight and height, the presence or absence of hypertension and diabetic mellitus, and any history of smoking (current, past, or nonsmoker) was also obtained. The differential diagnoses of tAMD and PCV were made in accordance with previous reports [10–12]. Briefly, the tAMD group included only wet tAMD with clear images of the vascular CNV networks on ICG. The PCV cases in the present study showed subretinal reddish-orange protrusions corresponding with the choroidal origin of the polypoidal lesions, typically with the vascular networks in the posterior poles on ICG [13, 14]. Patients with past histories of retinal vessel occlusion, uveitis, rhegmatogenous retinal detachment, or glaucoma were excluded. Patients who received previous photodynamic therapy (PDT) within 3 months or any other treatments within 6 months for AMD were also excluded.

All patients received 3 consecutive IVRs every month as previously described and followed up monthly for 3–12 months from the initial IVR [3, 4]. Additional IVR was performed as needed, namely, when sustained or recurrent serous retinal detachment, macular edema, or hemorrhage was recognized.

For statistical analysis, first, we compared the gender, age, BCVA, greatest linear dimension (GLD), central retinal thickness (CRT), history of smoking, presence of hypertension and diabetes mellitus, and body mass index (BMI) at baseline between the tAMD and PCV groups. Changes in the BCVA and CRT were then compared until 3 months after the initial IVR. Since the early response to IVR is known to be achieved within 3 months using monthly IVRs, we focused on the comparison between the effects of IVR in tAMD and PCV during this term. To evaluate the factors useful for predicting the BCVA at 3 months after the initial IVR, stepwise multiple regression analyses with backward elimination methods were performed using explanatory variables included gender, age, baseline BCVA, baseline GLD, baseline CRT, history of smoking, presence of hypertension and diabetes mellitus, BMI, and lesion phenotype (tAMD or PCV). The visual acuities were determined using a Landolt C chart and were converted to logarithm of the minimum angle of resolution (logMAR) values for calculation.

The stepwise multiple regression analysis was performed by IBM SPSS Statistics 19 software (IBM Inc., Armonk, NY, USA), and all other statistical analyses were performed by MedCalc v.11.3 software (MedCalc Software bvba, Mariakerke, Belgium). Except for the multivariate analysis, a two-tailed *t*-test or Wilcoxon's signed-rank test was performed to compare any two groups. *P* values of  .05 or less were considered to be statistically significant.

## 3. Results

**Figure 1:**
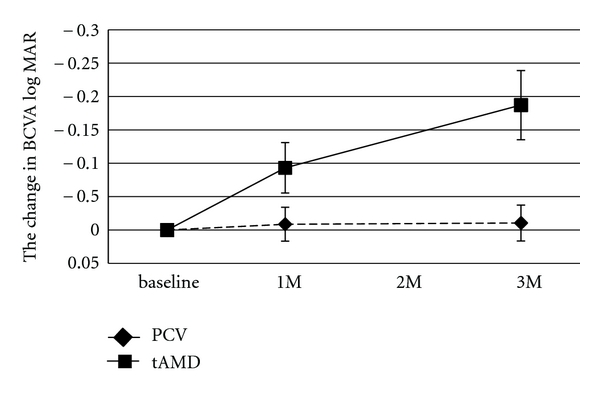
Changes in the best-corrected visual acuity (BCVA) for tAMD and PCV patients after intravitreal ranibizumab. The BCVA was determined using the Landolt C chart and was presented as decimal visual acuities. Diamonds with dashed lines: tAMD; squares with solid lines: PCV. Values represent means ± SEM.

**Figure 2:**
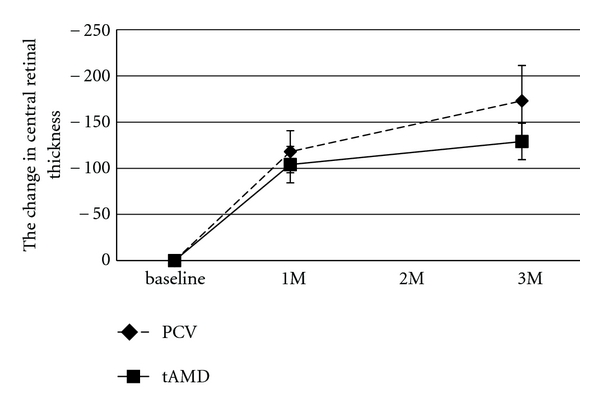
Changes in central retinal thickness (CRT) for tAMD and PCV patients after intravitreal ranibizumab. Diamonds with dashed lines: tAMD; squares with solid lines: PCV. Values represent means ± SEM.

The data summary of each phenotype (tAMD and PCV) is shown in Table 1. Patients with tAMD showed significantly better visual outcomes than patients with PCV. In the time course analysis, the BCVA at 1 month and 3 months after the initial IVR was significantly improved as compared with the baseline BCVA in the tAMD group (*P* = .0084 and *P* = .0008, resp., Wilcoxon's signed-rank test). In contrast, in the PCV group, the BCVA at 1 month and 3 months tended to improve over the baseline BCVA, but there was no statistical significance (*P* = .38 and *P* = .55, resp., Wilcoxon's signed-rank test) (Figure 1 and Table 2). The improvement of the BCVA at 3 months after the initial IVR from baseline was significantly greater in the tAMD patients than in the PCV patients (*P* = .0045; Mann-Whitney *U* test). In the 52 eyes (tAMD 27, PCV 25 eyes) which were followed-up for 12 months after the first IVR, the BCVA was kept better than baseline (−0.14 and −0.11 at 6 months and 12 months, resp.) in the tAMD group although the significance was reduced (*P* = .018 and 0.064 at 6 months and 12 months, resp.). In the PCV group, no significant improvement of BCVA was found up to 12 months after the first IVR (−0.03, *P* = .35 and −0.0047, *P* = .90 at 6 months and 12 months, resp.). In addition, BCVA in the group having pretreatment subretinal hemorrhage within 1 disc area was significantly improved 3 months after the first IVR whereas what in the group having subretinal hemorrhage more than 1 disc area was not improved at the same time period (Table 3). The time course of the mean CRT in the tAMD and PCV groups is shown in Figure 2. Both phenotypes showed significant improvements in the CRT during the 3 months after the initial IVR (Table 2). There were no significant differences in the improvements of the CRT in the tAMD versus the PCV group.

The results of the stepwise multiple regression analyses are shown in Table 4. Two factors were significantly associated with the change of BCVA at 3 months after the initial IVR, which were actually more improved in (i) patients with a worse pretreatment BCVA (*P* = .026) and (ii) tAMD lesions (*P* = .013).

As complications, slight lens damage was found in one case after the second IVR. No other ocular or systemic complications were detected during the follow-up term in the present study.

## 4. Discussion

We compared the early response to IVR in different phenotypes of exudative AMD and demonstrated that the visual improvement was significantly greater in tAMD subjects than in PCV. In other words, the phenotype of exudative AMD was a significant prognostic factor for the visual acuity after IVR.

Currently, IVR is the leading therapy for exudative AMD [15, 16], pathological myopia [17, 18], idiopathic CNV [19], and many secondary CNVs [19, 20]. Previous randomized studies with large populations have demonstrated the efficacy of IVR for exudative AMD with classic and occult CNV lesions found using FA in Caucasian populations [21, 22]. However, occult CNV lesions in Japanese exudative AMD, which likely includes a number of PCV patients, did not show as good response to intravitreal bevacizumab as Caucasian subjects [5, 8, 23]. PCV is known to have some different characteristics as compared with tAMD, such as orange-red protrusions at the posterior pole of the retina and distinct forms of choroidal vascular abnormalities, including vascular networks of choroidal origin with polypoidal lesions at their border visualized by ICG [11, 13]. PCV often shows spontaneous regression in its natural course, but on the other hand, it often causes severe hemorrhagic and exudative changes that result in a poor visual prognosis [12, 13]. PCV is known to have a better response to photodynamic therapy (PDT) than tAMD, but the reason for this is not understood [24, 25]. Since PCV accounts for 54.7% of patients with neovascular AMD in the Japanese population [6] and 22.3% in the Chinese population [26], it is important to determine if there are some differences in the efficacy of anti-VEGF therapy against PCV and tAMD to choose the correct interventions for neovascular AMD in Asian populations. 

Our results showed a significant increase in the mean BCVA in patients with tAMD and a modest improvement in those with PCV. It was interesting that PCV patients showed poorer improvements in their BCVA than tAMD patients, although both phenotypes showed similar significant improvements in their CRT during 3 months after the initial IVR. A previous report showed a decrease in macular edema after three monthly bevacizumab injections in PCV cases [27]. Similarly, macular edema evaluated by CRT measurements was improved in four out of five eyes with PCV (80%) in the PEARL study. However, the improvement in the BCVA was less than that in the ANCHOR trial or the MARINA trial, although the reasons are unknown [28]. We hypothesized that there might be factors other than macular edema which influence the visual acuity in tAMD and PCV cases differently. Although the mean baseline GLD was significantly greater in the PCV group than in the tAMD group in the present study, the results of multivariate regression analysis revealed that the lesion phenotype (tAMD or PCV) was the independent prognostic factor for the 3-month visual outcome after IVR. As described in previous reports, the baseline BCVA is another factor that may influence the early effects of IVR. We found that a reduction of BCVA improvement after the initial 3 monthly IVR was predominantly due to the recurrence of the lesions, which suggested that more frequent IVRs were required during the follow-up period. However, greater improvement of BCVA in the first 3 months indicated better visual prognosis in tAMD than PCV treated by IVR.

Currently, there are some discrepancies in the literature regarding the effects of IVR against PCV. Some studies reported that the polypoidal lesions of PCV were barely resolved by anti-VEGF monotherapy, which might explain the limited efficacy of IVR on PCV [8, 9]. However, other reports suggested that the disappearance of the polypoidal lesions occurred at a high rate in the PCV cases with anti-VEGF monotherapy [27, 29, 30]. Although the PCV cases showed a lower response (increased BCVA) to IVR than the tAMD patients during the first 3 months in the present study, several studies reported a significant improvement of the visual acuity in PCV patients using IVR without any comparison to that in tAMD patients [29, 30]. Moreover, several reports have shown different outcomes for photodynamic therapy (PDT) between tAMD and PCV [24, 25]. In those reports, significantly better visual outcomes in PCV than in tAMD were demonstrated. Hence, it is important to evaluate the long-term results of IVR with a large number of subjects to determine the efficacy and durability of this therapy, particularly in PCV patients. Taken together, further investigation will be needed to determine the correct indications of IVR for exudative AMD.

## 5. Conclusion

IVR is an effective therapy for prompt increases in the BCVA in tAMD patients, although this effect might be limited in PCV patients in a Japanese population.

##  Conflict of Interests

The authors declare that there is no conflict of interests.

## Figures and Tables

**Table 1:** Data summary of the participants stratified by AMD phenotype.

	PCV	tAMD	total	*P* value
	(*n* = 32)	(*n* = 28)	(*n* = 60)	
Right eyes (%)	15 (47%)	12 (43%)	27 (45%)	.96^†^
Males (%)	25 (78%)	23 (82%)	48 (80%)	.95^†^
Mean age (±SD)Years	74.6 (±9.0)	74.4 (±9.2)	74.5 (±9.0)	.93*
Baseline BCVA LogMAR (±SD)	0.46 (±0.31)	0.64 (±0.39)	0.55 (±0.36)	.053*
Baseline CRT (±SD) *μ*m	414 (±191)	374 (±109)	395 (±158)	.34*
Baseline GLD (±SD) *μ*m	4558 (±1712)	3530 (±996)	4138 (±1479)	.0068*
Subretinal hemorrhage (>1 disc)	9 (28%)	5 (18%)	14 (23%)	.34^†^
BMI	22.2 (±2.5)	21.2 (±2.5)	22.4 (±2.9)	.56*
Present or ever smoker	20 (63%)	21 (75%)	41 (68%)	.45^†^
Hypertension	14 (44%)	10 (36%)	24 (40%)	.71^†^
Diabetes mellitus	5 (16%)	6 (21%)	11 (18%)	.81^†^

GLD: greatest linear dimension, BCVA: best-corrected visual acuity, CRT: central retinal thickness, and BMI: body mass index. *Unpaired *t*-test; ^†^×2 test.

**Table 2:** Changes in the BCVA and CRT from baseline after IVR treatment in tAMD and PCV patients.

	1 month		*P* value	3 months		*P* value
	Mean	Median (Interquartile range)		Mean	Median (Interquartile range)	

BCVA						
PCV	−0.0086	0.00 (−0.090–0.00)	.38*	−0.011	0.00 (−0.12–0.030)	.55*
tAMD	−0.093	0.00 (−0.17–0.00)	.0084*	−0.19	−0.12 (−0.30–0.00)	.00080*
*P* value		.42^†^			.0045^†^	

CRT						
PCV	−118	−111 (−166–−32)	<.0001*	−173	−130 (−212–−63)	<.0001*
tAMD	−104	−84 (−147–−57)	<.0001*	−129	−97 (−182–−64)	<.0001*
*P* value		.80^†^			.58^†^	

BCVA: best-corrected visual acuity (LogMAR); CRT: central retinal thickness.

*Wilcoxon's signed-rank test (comparison to baseline).

^†^Mann-Whitney *U* test (comparison between PCV and tAMD at the same month).

**Table 3:** Changes in the BCVA from baseline after IVR treatment in the patients having subretinal hemorrhage before treatment.

	1 month (*n* = 60)		*P* value	3 months (*n* = 60)		*P* value
Subretinal hemorrhage	Mean	Median (Interquartile range)		Mean	Median (Interquartile range)	

>1 DA (*n* = 14)	0.0053	0.00 (−0.079–0.18)	.57*	−0.040	0.00 (−0.097–0.020)	1.00*
≦1 DA (*n* = 46)	−0.064	−0.023 (−0.12–0.00)	.0008*	−0.11	−0.097 (−0.18–0.00)	.0002*
*P* value		.065^†^			.10^†^	

BCVA: best-corrected visual acuity (LogMAR); DA: disc area.

*Wilcoxon's signed-rank test (comparison to baseline).

^†^Mann-Whitney *U* test (comparison between PCV and tAMD at the same month).

**Table 4:** Multiple regression analysis of preoperative variables with the changes in BCVA between the baseline and 3 months after the initial IVR.

Variable	*r*	*P* value
Gender	−0.19	.15
(female = 0, male = 1)		
Smoking	−0.14	.30
(nonsmoker = 0, present and ever smokers = 1)		
Hypertension	0.11	.41
(no history = 0, present = 1)		
Diabetes Mellitus	0.041	.76
(no history = 0, present = 1)		
Phenotype	−0.38	.013
(PCV = 0, tAMD = 1)		
Baseline BCVA (LogMAR)	−0.36	.026
Baseline CRT (*μ*m)	0.11	.40
Subretinal hemorrhage (≦1 DA = 0, >1 DA = 1)	0.21	.11
Greatest linear dimension (*μ*m)	0.15	.27

BCVA: best-corrected visual acuity, IVR: intravitreous ranibizumab, CRT: central retinal thickness, and DA: disc area. r: partial correlation coefficient.
